# The relationship between performance in a theory of mind task and intrinsic functional connectivity in youth with early onset psychosis

**DOI:** 10.1016/j.dcn.2019.100726

**Published:** 2019-11-05

**Authors:** Daniel Ilzarbe, Elena de la Serna, Inmaculada Baeza, Mireia Rosa, Olga Puig, Anna Calvo, Mireia Masias, Roger Borras, Jose C. Pariente, Josefina Castro-Fornieles, Gisela Sugranyes

**Affiliations:** a Institut d'Investigacions Biomèdiques August Pi i Sunyer (IDIBAPS), Barcelona, Spain; b Department of Child and Adolescent Psychiatry, 2017SGR881, Institute of Neurosciences, Hospital Clinic de Barcelona, Barcelona, Spain; c Department of Medicine, Universitat de Barcelona, Barcelona, Spain; d Department of Child and Adolescent Psychiatry, Institute of Psychology, Psychiatry and Neuroscience, King’s College London, London, United Kingdom; e Centro de Investigación Biomédica en Red Salud Mental (CIBERSAM), Madrid, Spain

**Keywords:** DMN, Default Mode Network, EOAff, early onset affective disorders, EOP, early onset psychosis, EOSz, early onset schizophrenia, fMRI, functional Magnetic Resonance Imaging, gIQ, Global Intelligence Quotient, ToM, theory of mind, Adolescent, Early onset psychosis, Theory of mind, Functional neuroimaging, Resting-state

## Abstract

Psychotic disorders are characterized by theory of mind (ToM) impairment. Although ToM undergoes maturational changes throughout adolescence, there is a lack of studies examining ToM performance and its brain functional correlates in individuals with an early onset of psychosis (EOP; onset prior to age 18), and its relationship with age. Twenty-seven individuals with EOP were compared with 41 healthy volunteers using the “Reading-the-Mind-in-the-Eyes” Test, as a measure of ToM performance. A resting-state functional MRI scan was also acquired, in which the default mode network was used to identify areas relevant to ToM processing employing independent component analysis. Group effects revealed worse ToM performance and less intrinsic functional connectivity in the medial prefrontal cortex in EOP relative to healthy volunteers. Group by age interaction revealed age-positive associations in ToM task performance and in intrinsic connectivity in the medial prefrontal cortex in healthy volunteers, which were not present in EOP. Differences in ToM performance were partially mediated by intrinsic functional connectivity in the medial prefrontal cortex. Poorer ToM performance in EOP, coupled with less medial prefrontal cortex connectivity, could be associated with the impact of psychosis during a critical period of development of the social brain, limiting normative age-related maturation.

## Highlights

• Youth with early-onset psychosis displayed deficits in theory of mind performance.
• They also showed reduced intrinsic connectivity in the medial prefrontal cortex.
• Differences in theory of mind were partially mediated by prefrontal connectivity.
• Both measures failed to show the age-positive associations observed in controls.
• Onset of psychosis in adolescence may impact development of social cognition.

## 1 Introduction

Social cognition refers to the ability of human beings to interact with others by recognizing their emotions and thoughts. Various psychological processes are considered to be involved in social cognition: facial emotion processing and “theory of mind” (ToM) are the most frequently studied, among others such us empathy, or humor (Uekermann et al., 2010). ToM (Premack and Woodruff, 1978) or *mentalization* (Frith et al., 1991) is the capacity of understanding that others present independent beliefs, intentions or desires, and of attributing their mental states to predict their reactions and behaviour. The processes involved in social cognition are considered to play a key role in successful social interactions (Wade et al., 2018; Yager and Ehmann, 2006). Deficits in ToM have been historically associated with autism spectrum disorders (Baron-Cohen et al., 1985; Yirmiya et al., 1998), however in the last decades it has been suggested that impairments in ToM also underlie social difficulties observed in other mental health conditions (Korkmaz, 2011). Several meta-analyses have confirmed impaired ToM in schizophrenia (Bora et al., 2009) and affective disorders (Bora et al., 2016; Bora and Berk, 2016), with possibly more severe deficits in the former (Mitchell and Young, 2016). Recent reports have shown that ToM presents the highest correlation with everyday functioning in schizophrenia; stronger than any other neurocognitive domain (Bora, 2017; Fett et al., 2011).

In typically developing individuals, ToM performance improves with age during childhood, and peaks during adolescence (Valle et al., 2015; Wellman et al., 2001; Wilde Astington and Gopnik, 1991). The medial prefrontal cortex and bilateral temporo-parietal junction, which are the areas of the brain which are most consistently activated during performance of tasks assessing ToM during functional resonance imaging scanning (Schurz et al., 2014), are considered to undergo important functional and structural changes during adolescence (Blakemore, 2008). A study in adults with schizophrenia has suggested that earlier onset of the disorder is associated with greater social cognitive deficits (Linke et al., 2015), suggesting that age of onset may modulate later cognitive function. Studies examining ToM in adolescents with early onset psychosis (EOP; when first psychotic episode takes place before age 18) (Bourgou et al., 2016; Korver-Nieberg et al., 2013; Li et al., 2017; Pilowsky et al., 2000; Tin et al., 2018), summarized in Table 1, have confirmed ToM deficits in this population, although they have reported either no effect of age on the findings or have failed to provide this information. A single study has examined the neural correlates of a social cognitive domain in individuals with EOP, in which the authors documented abnormal visual and facial emotion processing in relation to their healthy counterparts (Seiferth et al., 2009). However, to our knowledge, no studies so far have assessed the neural correlates of ToM in subjects with EOP.

**Table 1:** Summary of previous studies comparing performance in tasks assessing theory of mind in individuals with early onset psychosis relative to a control group.

Author and year	Sample	Age (years)	Sex (female)	Duration of disease (months)	ToM task	Results
Pilowsky et al., 2000***a***	12 EOSz	12.2 (SD = 1.7)	8%		Fact and value belief task, Deception task, False-belief task	Impaired ToM in EOP compared to HV in the false-belief task [No report on age effects]
	12 HV	8.5 (SD = 1.3)	25%			
Korver-Nieberg et al., 2013	32 EOP	17.1 (SD = 1.3)	39%		Perspective-taking task	No significant differences in cognitive ToM. No effect of age in the model.
	78 HV	16.3 (SD = 1.6)	36%			
Bourgou et al., 2016	12 EOSz	14.8 (SD = 1.7)	42%	30 ± 6	Moving Shapes Paradigm (*Frith-Happe* Animated Triangles)	Impaired ToM in EOP compared to HV. No correlation with age.
	12 HV	14.7 (SD = 1.5)	50%	–		
Li et al., 2017	35 EOSz	16.5 (SD = 1.4)	43%	16 ± 15	Yoni Task, Faux Pas Task	Impaired affective and cognitive ToM in EOP compared to HV. [No report on age effects]
	35 HV	16.3 (SD = 1.2)	43%	–		
Tin et al., 2018***b***	30 EOSz	17.5 (SD = 1.2)	37%	27 ± 16	Yoni Task, Faux Pas Task	Impaired affective and cognitive ToM in EOP compared to HV. [No report on age effects]
	30 HV	17.2 (SD = 1.0)	30%	–		

*Note*: HV = Healthy Volunteers; EOP = Early Onset Psychosis; EOSz = Early Onset Schizophrenia; ToM = theory of mind; ***a***: A third group with Autism Spectrum Disorder (n = 12; 8% female) also included for comparison; ***b***: A third group with Autism Spectrum Disorder (n = 30; 23% female) also included for comparison.

In adult patients with schizophrenia, resting-state connectivity (hereafter referred to as “intrinsic connectivity”) between areas of the brain which are typically recruited during performance of ToM tasks, has been found to be decreased (Schilbach et al., 2016). This network of brain areas overlaps with the Default Mode Network (DMN) (Schilbach et al., 2012), which conforms a set of brain regions which activate together during rest and which usually deactivate during goal-directed tasks (Greicius et al., 2003). In addition, intrinsic functional connectivity of the DMN has been related with ToM abilities in both healthy individuals (Li et al., 2014) and in adults with schizophrenia (Zemánková et al., 2018). In fact, intrinsic connectivity has been suggested to be a better predictor of social functioning and cognitive performance than task-based functional Magnetic Resonance Imaging (fMRI) (Viviano et al., 2018).

There is a lack of consensus concerning changes in connectivity of the DMN characterizing psychotic samples: a small number of studies have reported over-connectivity of the DMN during the resting-state in psychosis (Tang et al., 2013), while a recent meta-analysis has documented less intrinsic functional connectivity within the DMN in both schizophrenia (Dong et al., 2018; Kühn and Gallinat, 2013) and bipolar disorder with psychotic features (Syan et al., 2018). In contrast, some studies have reported less connectivity in schizophrenia compared to bipolar disorder, regardless of psychotic symptoms, in which values of intrinsic functional connectivity were intermediate relative to controls (Argyelan et al., 2014; Öngür et al., 2010; Skåtun et al., 2016), while others have found similar deficits in both conditions (Khadka et al., 2013).

From a developmental perspective, a recent meta-analysis focusing on the DMN in healthy individuals has demonstrated greater connectivity in adults compared to children (Mak et al., 2017). The single study reporting an effect of age on seed-based connectivity of the DMN in participants with a first episode of early-onset schizophrenia (Jiang et al., 2015) reported that intrinsic connectivity between a seed located in the precuneus and the left inferior frontal cortex was positively associated with age, while connectivity between a seed in the left middle occipital cortex and the precuneus was negatively associated with age. In contrast, there were no age effects in intrinsic connectivity of the DMN in patients with an adult onset of schizophrenia (Jiang et al., 2015). This study excluded subjects with affective psychosis and did not explore the association between brain imaging measures and social cognitive performance.

In this context we set out to evaluate performance during a ToM task and its relationship with intrinsic functional connectivity during resting-state fMRI, in individuals with EOP compared to healthy volunteers, and to examine the effect of age on these measures. Our hypotheses were: 1) patients with EOP would display worse ToM performance than healthy volunteers; 2) patients with EOP would exhibit less intrinsic functional connectivity within the DMN compared to healthy volunteers; 3) patients with EOP would fail to display the age-related improvements in ToM performance and increases in DMN connectivity exhibited by healthy volunteers; and 4) Differences in ToM performance would be mediated by intrinsic functional connectivity within the DMN.

## 2 Materials and methods

This is a cross-sectional case-control study carried out at the Department of Child and Adolescent Psychiatry and Psychology of Hospital Clinic of Barcelona (Spain), approved by the local Ethical Review Board.

### 2.1 Sample

Twenty-seven participants with EOP were consecutively included. Diagnosis of first episode of psychosis was established at first contact with mental health services and defined as the presence of positive psychotic symptoms of less than 6 months duration with an onset between the ages of 12 and 17 (for details on baseline recruitment and assessment see Castro-Fornieles et al., 2007). Exclusion criteria consisted of: 1) presence of a concomitant disorder that could account for the psychotic symptoms such as autism spectrum disorders, post-traumatic stress disorder or drug-induced psychoses (occasional substance use was not an exclusion criterion); 2) intellectual disability according to DSM-IV-TR criteria; 3) neurological disorders or history of head trauma with loss of consciousness; 4) pregnancy and 5) medical or technical counterindications for the MRI (i.e. metal implants, brain aneurysms, etcetera).

For the current study, all individuals with EOP were assessed 2 years after the diagnosis of their first episode of psychosis; thus, duration of disease was homogeneous and current age and age at onset were highly correlated (r = .98; *p* <  .0001). Forty-one age and sex matched healthy volunteers were recruited from schools or community settings from the same geographical area as individuals with EOP. Additional exclusion criteria for healthy volunteers were as follows: 1) any current or life-time Axis I disorder; 2) any psychotic disorder in 1^st^ and 2^nd^ degree relatives. All participants provided written informed assent, and parents or legal guardians gave written informed consent before the study began.

### 2.2 Clinical assessment

Demographic data, including age, sex and race, was collected; socio-economic status was classified according to the Hollingshead-Redlich scale (Hollingshead AB, 2007), where the highest parental educational and employment status was recorded.

All participants were assessed 2 years after the first episode by mental health professionals (psychiatrists and psychologists) with experience diagnosing and evaluating children and adolescents with semi-structured interviews, clinical scales and neuropsychological tests. Diagnoses were re-assessed using the Kiddie-Schedule for Affective Disorders and Schizophrenia, Present and Lifetime version (Kaufman et al., 1997) in its Spanish version (Ulloa et al., 2006) according to DSM-IV-TR criteria (American Psychiatric Association, 2000). Clinical severity in individuals with EOP was evaluated using the Positive and Negative Syndrome Scale (PANSS), which is a 30-item scale organized in 3 subscales: positive and negative symptoms and general psychopathology; with each item scored between 1 and 7, from absent to extreme (Kay et al., 1987). Detailed medication history was recorded for each participant; doses of antipsychotic drugs were transformed into chlorpromazine equivalents (Leucht et al., 2014) and cumulative chlorpromazine equivalents over time were calculated for each individual at the moment of scanning.

Theory of mind was evaluated using the child version (Baron-Cohen et al., 2001a) of the “Reading-the-Mind-in-the-Eyes” Test (Baron-Cohen et al., 2001b), which presents 28 images of multiple expressions of different subjects’ eyes. It includes a control condition, where participants are asked to identify the sex of poser, and an experimental condition testing emotion identification between a 4-option-multiple choice question.

Neurocognitive level was measured using the Vocabulary, Similarities, Block Design and Matrix Reasoning subtests of the Spanish version of the Wechsler Intelligence Scale for Children - Fourth Edition (WISC-IV) (Wechsler, 2003) or Wechsler Adult Intelligence Scale–III, revised (Wechsler, 2011). The General Ability Index (referred to as Global Intelligence Quotient; gIQ), derived from the Verbal Comprehension and Perceptual Reasoning indices, was used as an index of intelligence level (Flanagan and Kaufman, 2008).

### 2.3 Statistical analyses

Statistical analysis was performed in Stata v.13.1 using *t*-test and chi-square for demographic and clinical information. Behavioural performance during the ToM task was compared using multilevel mixed-effects linear regression models with group, condition and group by condition interaction as fixed effects and including individual factor as random effect; applying Bonferroni correction for multiple pairwise comparisons. gIQ, sex, age, socio-economic status and group by age interaction were added as covariates when achieving significance level *p* ≤  .05. Linear regression models for each condition, control and experimental, were built for assessing the effect of age on behavioural measures including the group by age interaction. Again, gIQ, socio-economic status and sex were added as covariates when significant (*p* ≤  .05). Effect sizes were calculated for significant post-hoc paired *t*-tests (Cohen’s *d*) and linear regression models (*ω^2^*). Within the EOP group, additional analyses were conducted to assess the relationship between symptom severity and age, and the effect of symptom severity on ToM performance.

### 2.4 Neuroimaging acquisition

An 8-min resting-state fMRI sequence was acquired on a 3 T Siemens Magnetom Trio Tim (Siemens Medical Systems, Germany) scanner at the Magnetic Resonance Image Core Facility of IDIBAPS, Centre for Image Diagnosis, Hospital Clínic of Barcelona. Participants were instructed to keep their eyes closed, remain as still as possible for the duration of the scanning session. A technician engaged in conversation with the participant before and after the resting-state session to guarantee that they did not fall asleep. Acquisition parameters were as follows: 240 volumes, TR = 2000 ms; TE = 29 ms; matrix size = 480 × 480; slice thickness = 4 mm, acquisition matrix = 80 × 80 mm, 32 slices, voxel size 3 × 3 × 4 mm.

### 2.5 Neuroimaging preprocessing

A DARTEL algorithm was applied to the segmented T1-structural volumes to generate a sample-specific template. Resting-state fMRI images were realigned, co-registered to the individual T1-weighted scan (segmented using the sample specific template), normalized to the Montreal Neurological Institute (MNI) space and smoothed using a 6-mm Gaussian kernel in SPM12. One healthy volunteer and three participants with EOP were excluded from further neuroimaging analyses due to excessive motion (mean Framewise Displacement >.2 mm) (Power et al., 2017, 2012; Yan et al., 2013); these individuals did not differ in age, sex or socio-economic status from those included in the analysis (*p*s ≥ .31).

### 2.6 Functional connectivity analysis

The component corresponding to the DMN was identified with independent component analysis using the GIFT toolbox v3.0b for SPM12 running on Matlab R2017b. Independent component analysis decomposes fMRI data into spatially independent patterns, which include both functional networks and sources of noise (such as motion or cerebrospinal fluid), thus allowing to minimize the influence of artefact on the findings (Pruim et al., 2015; Salimi-Khorshidi et al., 2014). Furthermore, the fact that it is a data-driven approach, which allows to avoid the potential bias of pre-determined regions of interest, is an additional advantage given the novelty of the study design. The DMN was identified by visual inspection and confirmed through the highest correlation with the template (r = .61), and included the prefrontal cortex, precuneus and bilateral temporo-parietal junction [spatial map representation in figure A1 of appendix]. The spatial maps of the DMN component of each subject were compared in a whole brain *t*-test analysis in SPM, introducing group, age, group by age interaction and sex as regressors within an inclusive DMN mask created with the mean sample template. Only results surviving family-wise error correction are reported. Next, mean values of intrinsic functional connectivity within each significant cluster were extracted for each individual, and linear regression models were conducted in Stata v.13.1, in which the effects of gIQ, sex, age and socio-economic status were examined. These covariates were included in the model when significant (*p* ≤  .05). The potential effect of antipsychotic medication and symptom severity on resting-state fMRI measures (cumulative chlorpromazine equivalents; Leucht et al., 2014) was also evaluated within the EOP group. In order to assess whether the differences in ToM performance between healthy volunteers and EOP were associated with intrinsic functional connectivity within the DMN, a mediation analysis was carried out for clusters showing age-associated differences. The proportion of total effect mediated by functional connectivity was calculated based on standardised beta-values obtained from linear regression models.

For secondary analyses, cases were classified according to diagnosis at two-year assessment into early onset schizophrenia (EOSz) and early onset affective disorders (EOAff). Group, and group by age effects in ToM performance and intrinsic functional connectivity within the DMN were tested in these subgroups [See Supplementary Material].

## 3 Results

### 3.1 Sample

Socio-demographic and clinical information are presented in Table 2. There were no group differences in age, sex or race distribution. Individuals with EOP showed significantly lower gIQ (*p =* .0005) and socio-economic status (*p* = .017) than healthy volunteers. Diagnoses at 2 years within the case group were: schizophrenia (n = 9), schizoaffective disorder (n = 7), major depressive disorder with psychotic features (n = 3), bipolar spectrum disorders (bipolar I, n = 4; bipolar no otherwise specified, n = 2) and psychosis not otherwise specified (n = 2).

**Table 2:** Socio-demographic and, clinical characteristics of the sample.

	HV (n = 41)	EOP (n = 27)	*p* value
**Socio-demographic**			
Age (years) [range]	17.8 (SD = 1.6) [15.0–20.9]	18.1 (SD = 1.6) [15.7–20.1]	*.374*
Sex (% female)	56.1%	59.3%	*.796*
Race (% caucasian)	92.7%	81.5%	*.161*
Socio-economic Status	48.9 (SD = 16.0)	39.1 (SD = 15.0)	.017*
**Clinical variables**			
Global Intelligence Quotient	104.1 (SD = 9.8)	92.8 (SD = 15.7)	.0005*
PANSS (total score)	–	49.6 (SD = 16.2)	–
- Positive Subscale	–	10.1 (SD = 3.8)	–
- Negative Subscale	–	15.4 (SD = 6.5)	–
- General Subscale	–	24.4 (SD = 8.6)	–
Age of onset (years)	–	15.9 (SD = 1.5)	–
Duration of disease (months)	–	27 (SD = 3)	–

*Note*: HV = Healthy Volunteers; EOP = Early Onset Psychosis; PANSS = Positive and Negative Syndrome Scale; SD = Standard Deviation; * *p* < .05.

### 3.2 Theory of mind task

**Fig. 1:**
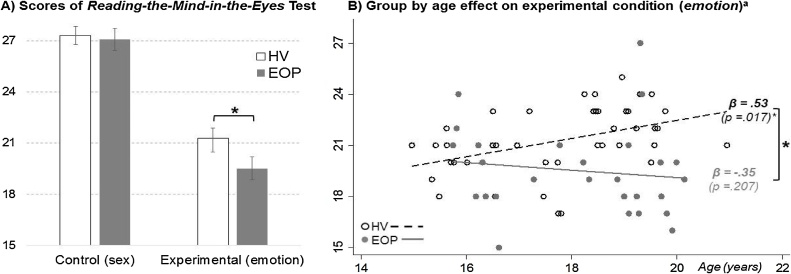
Bar graphs representing mean least squares (95% confidence intervals) of performance in the control and experimental conditions of the “Reading-the-Mind-in-the-Eyes” Test (A) and group by age effect on experimental condition (B) for the healthy volunteer (n = 41) and early onset psychosis groups (n = 27). Note: HV = Healthy Volunteers; EOP = Early Onset Psychosis; ^a^: model also including global intelligence quotient (*p* = .052) as covariable; * *p* < .05.

There were significant group by condition (*X*^2^ = 6.8; *p* = .009), group (*X*^2^ = 10.2; *p* = .001) and condition (*X*^2^ = 533.2; *p* < .0001) effects. Post-hoc analysis revealed significant differences in the experimental condition of the “Reading-the-Mind-in-the-Eyes” Test (*p* < .001; Cohen’s *d* = .79), whereby individuals with EOP showed poorer performance, while no between group differences were observed in the control condition (*p* = 1.0) [Fig. 1A]. Linear regression models showed a significant group by age effect only in the ToM condition (*p* = .014; *ω^2^* = .21), where ToM scores and age were positively associated in healthy volunteers (β = .53; *p* = .017), but not in the EOP group (β = −.35; *p* = .207) [Fig. 1B]. gIQ was included as covariate only in the linear regression model (*p* = .052); sex and socio-economic status had no significant effect in either model. A negative correlation was found between the total PANSS score and age of illness onset (r = −.44; *p* = .02). Therefore, within the EOP group, the effect of severity of symptoms (PANSS: total score and subscales) on the age by ToM performance model was tested; these analyses failed to achieve significance (βs ≤ |.13|; *ps* ≥ .13).

### 3.3 Intrinsic functional connectivity

**Fig. 2:**
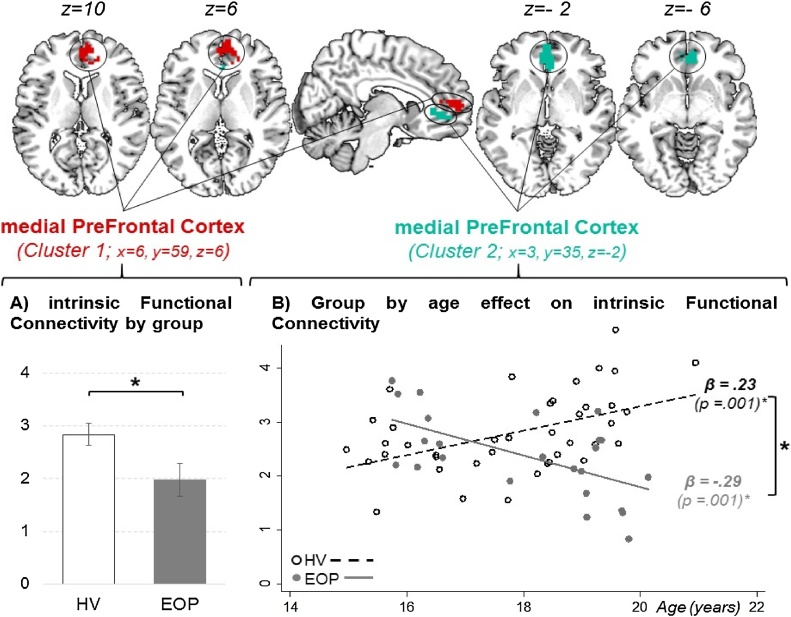
Clusters within the Default Mode Network showing significant group effect (A) and group by age interaction (B) in intrinsic functional connectivity between participants with early onset psychosis (n = 24) compared to healthy volunteers (n = 40). Note: HV = Healthy Volunteers; EOP = Early Onset Psychosis; * *p* < .05.

During resting-state fMRI, there was an effect of group in the medial prefrontal cortex within the DMN (cluster 1: [x = 6, y = 59, z = 6]; voxel count = 54; *p*^FWE−corr^ = .036), whereby EOP participants exhibited less connectivity compared to healthy volunteers [Fig. 2A]. A second cluster in the medial prefrontal cortex showed a significant group by age interaction (cluster 2: [x = 3, y = 35, z = −2]; voxel = 66; *p*^FWE−corr^ = .017): whereby connectivity was positively associated with age in HV (β = .23; *p* = .001), while the effect was the opposite in individuals with EOP (β = −.29; *p* = .001) [Fig. 2B]. There was no significant effect of socio-economic status, sex or gIQ for either of the clusters, thus these factors were excluded from the model. Within the EOP group, severity of symptoms (PANSS: total score and subscales) showed no significant effect when introduced in the model assessing age in cluster 2 (βs ≤ |.03|; *ps* ≥ .16). In individuals with EOP, cumulative chlorpromazine equivalents were not correlated with the mean extracted values of intrinsic functional connectivity in either of these clusters (*r*s ≤ |.05|; *ps* ≥ .83).

**Fig. 3:**
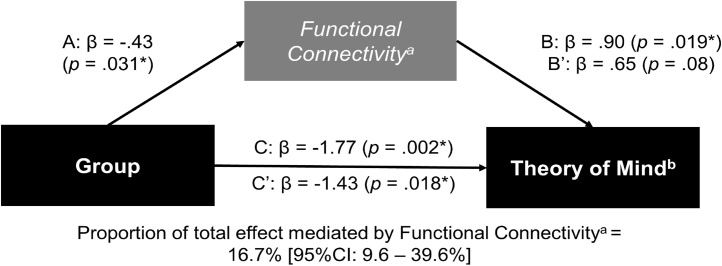
Mediation analysis illustrating the relationship between intrinsic functional connectivity in the medial Prefrontal Cortex, within the Default Mode Network, with performance in the “Reading-the-Mind-in-the-Eyes” Test. Note: ^a^ = Functional connectivity in the medial Prefrontal Cortex (cluster 2; [x = 3, y = 35, z = −2]); ^b^ = performance in the experimental condition of the “Reading-the-Mind-in-the-Eyes” Test; * *p* < .05.

Mediation analysis showed that intrinsic functional connectivity in the medial prefrontal cortex (cluster 2) within the DMN accounted for 16.7% (95%IC: 9.6%–39.6%) of the differences in ToM performance exhibited by the participants with EOP [Fig. 3; table A1 of the Supplementary Material].

Secondary analyses, dividing the sample by diagnostic groups, showed that only participants with EOSz exhibited impaired performance during the “Reading-the-Mind-in-the-Eyes” Test compared to EOAff and healthy volunteers (*p*s ≤ .008; Cohen’s d ≥ |1.03|). In contrast, group differences in intrinsic functional connectivity in cluster 1 (*p*s ≤ .005) and the group by age interaction in cluster 2 (*p*s ≤ .001) remained significant for both patient subgroups compared to healthy volunteers [see table A2 and figures A3-A4 in Supplementary Material].

## 4 Discussion

Our study evaluating ToM performance and resting-state fMRI in individuals with EOP has found that:

(1) Patients with EOP performed significantly worse than healthy volunteers in a task assessing ToM. There was a positive association between task performance and age in healthy volunteers, which was absent in individuals with EOP.
(2) Patients with EOP exhibited less intrinsic connectivity in the DMN, specifically in the medial prefrontal cortex, than healthy volunteers. Connectivity in this region and age were positively associated in healthy volunteers and negatively associated in EOP.
(3) Differences in performance in the ToM task were partially mediated by intrinsic functional connectivity in the medial prefrontal cortex within the DMN.
(4) Patients with EOSz performed significantly worse than individuals with EOAff and than healthy volunteers in the ToM task, while there were no differences between diagnostic groups in DMN connectivity.

In this sample of patients with EOP, we observed worse performance in a task assessing ToM in patients relative to healthy volunteers, despite controlling for differences in global intelligence between groups. Our findings concerning ToM impairment in individuals with EOP are in line with both a meta-analysis of adult samples with schizophrenia (Bora et al., 2009), and several studies encompassing youth with EOP (Bourgou et al., 2016; Korver-Nieberg et al., 2013; Li et al., 2017; Pilowsky et al., 2000; Tin et al., 2018). Our results reflect both a cross-sectional deficit and lack of age-related gain in ToM performance in EOP compared to healthy volunteers. Similar to our findings, the few studies evaluating ToM in participants with EOP so far have also failed to observe a contribution of age on ToM performance in EOP patients (Bourgou et al., 2016; Korver-Nieberg et al., 2013; Pilowsky et al., 2000), nor was this observed in a meta-regression with age of onset in a meta-analysis of adult schizophrenia samples (Bora et al., 2009). In addition, we ruled out that greater symptom severity, associated with earlier onset of psychosis in ours and other samples (Vyas et al., 2011), could have contributed to the ToM deficits documented in our sample. In this regard, a recent meta-analysis assessing performance in the “Reading-the-Mind-in-the-Eyes” Test in individuals with autism spectrum disorders showed that ToM scores were positively correlated with age in the control group (Peñuelas-Calvo et al., 2018) -similar to our findings in healthy volunteers-, but not in the group with autism spectrum disorders. Of note, similar performance deficits have been reported in the “Reading-the-Mind-in-the-Eyes” Test between adults with autism spectrum disorders and with schizophrenia (Couture et al., 2010; Craig et al., 2004; Lugnegård et al., 2013; Murphy, 2006). Despite the cross-sectional design, our results point towards a potential developmental discontinuation in the acquisition of ToM skills in EOP. Although the current study design does not shed light on the timing of this process, previous studies have documented ToM impairments in individuals at ultra-high risk for psychosis, who displayed intermediate performance between healthy controls and individuals with schizophrenia (Bora and Pantelis, 2013); reflecting that social cognitive deficits may have an onset prior to clinical disease (Zhang et al., 2018). The emergence of prodromal symptoms and/or a psychotic disorder during adolescence, coinciding with the time in which social cognition and function consolidate (Valle et al., 2015; Wellman et al., 2001), is likely to have an impact on ToM performance. In sum, ToM impairment in individuals with EOP, which appears to be independent of symptom severity or deficits in global intelligence, could be related to a lack of developmental gain in abilities usually acquired during childhood and adolescence, and should be taken into account when tailoring interventions for youth with an EOP (Turner et al., 2018).

We observed less intrinsic functional connectivity in the medial prefrontal cortex within the DMN in individuals with EOP, which is in line with findings from a meta-analysis of resting-state fMRI studies in schizophrenia (Dong et al., 2018; Kühn and Gallinat, 2013). Studies of resting-state fMRI in typically developing youth have shown that networks are built from early childhood: global efficiency and the strength of intrinsic functional connectivity within networks increase over development, with a mean maximum connectivity at age 22 years (Cai et al., 2018; Dosenbach et al., 2010). Specifically, the medial prefrontal cortex is the region with the greatest number of connections correlating with age in the DMN (Sato et al., 2014). Our cross-sectional study of adolescents and young adults up to 20 years of age confirms this increase in connectivity within the DMN in typically developing youth, in contrast to youth with EOP, in whom intrinsic functional connectivity in the medial prefrontal cortex was negatively associated with age. The only study reporting a significant effect of age in functional connectivity in EOP to date employed a node-based analysis, and found that in patients with early onset schizophrenia, connectivity decreased with age between the occipital cortex and the left precuneus, while it increased between the right precuneus and inferior frontal gyrus, which contrasted with findings in healthy controls (Jiang et al., 2015). These results support the notion that abnormal age-related changes in brain functional connectivity may characterize youth with early onset schizophrenia, however the different methodological approach makes it difficult to directly compare to our study. The combination of cross-sectional deficits, together with age-negative associations in connectivity, raises the possibility that illness effects may play a role in the loss of previously developed connections. However, taking into account that duration of illness was similar across all participants, our findings of less hypoconnectivity within the DMN in younger EOP could also support the possibility of greater plasticity or capacity to recover from illness-related disruption at earlier ages. Together with the fact that the medial prefrontal cortex has been shown to be especially sensitive to developmental deviation, our findings support the view that medial prefrontal DMN connectivity may be more responsive to intervention at younger ages.

Our findings suggest a partial contribution of intrinsic functional connectivity in the medial prefrontal cortex to differences in ToM performance. Connectivity between the medial prefrontal cortex and temporo-parietal junction has been reported to play a role during ToM in task-based fMRI studies (Li et al., 2014); and hypoconnectivity of these brain regions has been described in resting-state fMRI studies in schizophrenia (Schilbach et al., 2016). ToM is one of the most consistent and stable dimensions identified during mind-wandering, which is considered to take place during the resting-state (Diaz et al., 2013). Several studies have documented significant correlations between ToM performance and connectivity during resting-state fMRI in adults with schizophrenia (Choe et al., 2018; Erdeniz et al., 2017; Mothersill et al., 2017; Zemánková et al., 2018). In a study in patients with chronic schizophrenia, performance in the “Reading-the-Mind-in-the-Eyes” Test positively correlated with connectivity between the left precuneus and right middle cingulate/right inferior frontal gyrus, and between the left temporo-parietal junction and right calcarine gyrus/right lingual gyrus; and negatively correlated with connectivity between the left precuneus and right insula and left superior temporal gyrus (Mothersill et al., 2017). Zemankova et al. also reported that empathy scores were positively and negatively associated with functional connectivity between the medial prefrontal cortex and other frontal regions in patients with schizophrenia, while they observed no significant association between affective ToM scores and functional connectivity in the medial prefrontal cortex in healthy volunteers (Zemánková et al., 2018). Our findings extend this evidence to a younger population, nearer to illness onset, and add to the notion that connectivity of the medial prefrontal cortex may exert an influence on ToM performance in EOP. Brain-based measures are likely to be more sensitive to biological processes underpinning psychosis than cognitive tasks, therefore suggesting a potential role for DMN connectivity as treatment target and/or means for monitoring treatment response in individuals with EOP.

While both patient groups exhibited reduced intrinsic functional connectivity within the DMN compared to healthy volunteers, only EOSz exhibited impaired performance during the “Reading-the-Mind-in-the-Eyes” Test. A majority of comparative studies have shown greater ToM impairment in schizophrenia relative to bipolar disorder (Caletti et al., 2013; Guastella et al., 2013; Thaler et al., 2013), in line with our results. Previous studies have supported that hypo-connectivity within the DMN may be specific to schizophrenia (Dong et al., 2018; Kühn and Gallinat, 2013) and to bipolar disorder with psychotic features in adults (Brady et al., 2017; Khadka et al., 2013; Meda et al., 2016), and adolescents (Zhong et al., 2018). In this context, our findings add support that hypo-connectivity of the DMN, specifically in the medial prefrontal cortex, may form part of a psychosis phenotype common to both affective and non-affective presentations of psychotic disorders, while ToM impairment may be specific to schizophrenia spectrum disorders.

The main limitation of our study is the sample size, especially in the secondary analyses presented in supplementary material when subdividing the EOP group by schizophrenia spectrum disorders and affective disorders, which may have resulted in lower statistical power, therefore limiting our capacity to detect statistically significant findings. However, the fact that the sample is composed of an understudied population –EOP-, and that it is homogeneous and clinically well characterised - all individuals have been followed-up since illness onset-, must also be taken into account when assessing the characteristics of the study. Although patients had a short illness duration, we cannot fully rule out that ToM deficits and lower connectivity of the DMN result solely from processes exerting an effect after illness onset, and not surrounding the illness onset. The fact that we do not find a relationship with exposure to antipsychotic medication, for example, argues against this; however, this should ideally be examined in a prospective design including pre-clinical adolescent cases. In contrast, the evaluation of participants 2 years after the first episode of psychosis has the advantage of capturing clinical diagnosis with greater stability (Castro-Fornieles et al., 2011), allowing to sub-classify the sample of EOP patients into schizophrenia and affective spectrum disorders. With regards the technique, the risk of sleep drifts are intrinsic to resting-state fMRI acquisition (Tagliazucchi and Laufs, 2014), although measures were put in place in order to minimize this. As mentioned, this is not a task-based fMRI study, thus clinical and neuroimaging correlations should be taken cautiously. However, it is worth noting that the cluster of hypoconnectivty we have found in the medial prefrontal cortex overlaps with a cluster identified in another study of resting-state fMRI in schizophrenia, in which regions-of-interest were selected according to their overlap between the DMN and brain areas recruited during tasks assessing social cognition (including ToM and excluding emotion recognition), in task-based fMRI designs (Schilbach et al., 2016, 2012). On the other hand, resting-state fMRI carries a number of advantages in relation to replicability (simpler instructions and less potential confounders), making it more comparable with other studies and easier to translate to clinical daily practice, especially considering cost and equipment requirements (Fox and Greicius, 2010). This is particularly relevant when considering the feasibility of scanning youth with EOP. Furthermore, one study has demonstrated that resting-state connectivity has shown to predict social functioning and cognitive performance better than task-based fMRI in schizophrenia (Viviano et al., 2018). Moreover, a study evaluating social skills training in adults with schizophrenia showed a correlation between improvement in social cognitive performance and connectivity of the DMN (Sestini et al., 2016), suggesting that specific interventions in patients with psychosis may have an impact on their social functioning which could potentially be mediated by changes in the underlying neural correlates of social cognition.

### 4.1 Conclusions

To conclude, our study provides evidence of ToM impairments and less intrinsic connectivity in the DMN in youth with EOP, and a lack of the age-positive or presence of age-negative association in each domain, in contrast to observations in healthy volunteers. Our data increases understanding of the neural underpinnings of social cognitive deficits in psychotic disorders, suggesting medial prefrontal cortex, within DMN connectivity, as a potential brain-based marker for identifying and monitoring social cognitive deficits. It also provides a plausible explanation for reports of greater social cognitive deficits in patients with an earlier age of onset of psychosis, suggesting the need to prioritize interventions targeting social cognition during adolescence.

## Disclosures and acknowledgements

DI has received funding from the Spanish Ministry of Science, Innovation and Universities, Instituto de Salud Carlos III, ‘Rio Hortega’ contract CM17/00019, with the support of European Social Fund), and a grant from the Alicia Koplowitz Foundation, as well as honoraria and travel support from Otsuka-Lundbeck and Janssen. ES has received research funding from the Instituto de Salud Carlos III. IB has received research funding from the Instituto de Salud Carlos III and the Alicia Koplowitz Foundation, and reports honoraria and travel support from Otsuka-Lundbeck and Janssen. OP has received research funding from the Alicia Koplowitz Foundation. JC has received research funding from the Instituto de Salud Carlos III, the Government of Catalonia, La Marató TV3 and the Alicia Koplowitz Foundation. GS has received research funding from the Brain and Behaviour Research Foundation (NARSAD Young Investigator Award), the Instituto de Salud Carlos III, the Government of Catalonia and the Alicia Koplowitz Foundation, and has received honoraria and travel support from Otsuka-Lundbeck, Janssen and Adamed Pharma. The remaining authors declare no conflicts of interest. This work was supported by Institut d'Investigacions Biomèdiques August Pi i Sunyer *IDIBAPS Start-up grant*) and Spanish Ministry of Economy and Competitiveness/Instituto de Salud Carlos III (PI18/00976), co-financed by ERDF Funds from the European Commission (“*A way of making Europe*”).

## Declaration of Competing Interest

No conflicts declared.
