# Supplementary material for: The relationship between performance in a theory of mind task and intrinsic functional connectivity in youth with early onset psychosis
Source: Dev Cogn Neurosci. 2019 Nov 5;40:100726. doi: 10.1016/j.dcn.2019.100726 (PMC6974903; doi:10.1016/j.dcn.2019.100726)
Supplement: Supplementary file 1 [file mmc1.docx]

*Daniel Ilzarbe, Elena de la Serna, Immaculada Baeza, Mireia Rosa, Olga Puig, Anna Calvo, Mireia Masias, Roger Borras, Jose C. Pariente, Josefina Castro-Fornieles and Gisela Sugranyes.* **The relationship between performance in a theory of mind task and intrinsic functional connectivity in youth with early onset psychosis**

Appendix A. Supplementary Data.

**Figure A1.** Spatial map of the Default Mode Network for the whole sample (overlaid on a grayscale brain template).


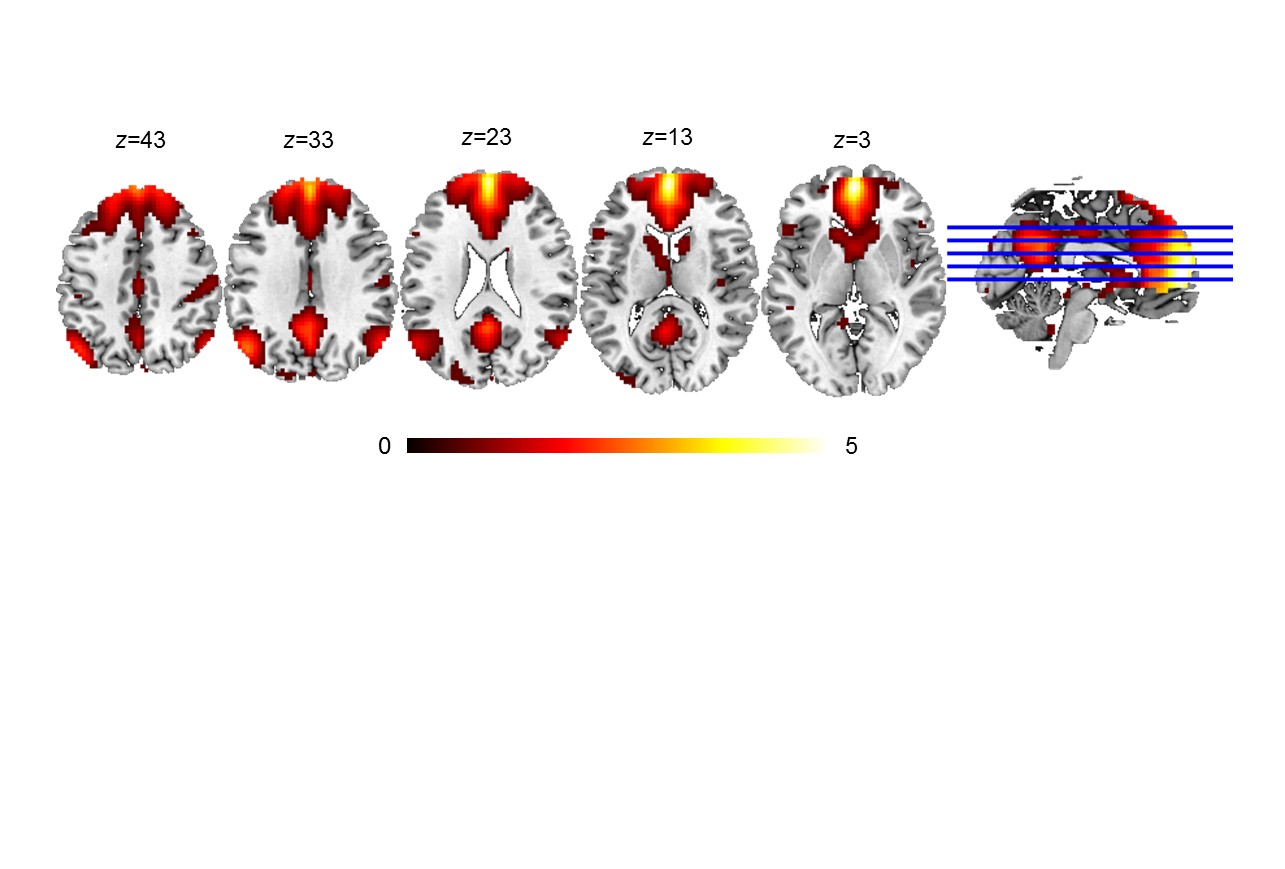


**Table A1.** Model testing the mediating effect of intrinsic functional connectivity in the medial Prefrontal Cortex on the relationship between group and performance in the “Reading-the-Mind-in-the-Eyes” Test

| Testing Paths | Beta | SE (Beta) | 95%CI | Beta (Standardized Coefficients) |
| --- | --- | --- | --- | --- |
| Path C: Theory of Mind (total score in the experimental condition of the “Reading-the-Mind-in-the-Eyes” Test | | | | |
| R^2^ = .13; F(1, 66) = 10.15; *p* = .002 | | | | |
| Dependent variable: Group | -1.77 | .56 | (-2.89; -.66) | -.37 |
| Path A: Functional Connectivity in medial Prefrontal Cortex within Default Mode Network (Cluster 2) | | | | |
| R^2^ = .07; F(1, 62) = 4.89; *p* = .03 | | | | |
| Dependent variable: Group | -.43 | .19 | (-.81; -.04) | -.27 |
| Path C’: Theory of Mind (total score in the experimental condition of the “Reading-the-Mind-in-the-Eyes” Test | | | | |
| R^2^ = .17; F(1, 61) = 6.09; *p* = .004 | | | | |
| Dependent variable: Group | -1.43 | .59 | (-2.61; -.25) | -.29 |
| Mediation variable: Functional Connectivity in medial Prefrontal Cortex within Default Mode Network (Cluster 2) | .65 | .37 | (-.09; 1.40) | .21 |
| Total (a) * (b’) |  |  |  | -.06 |

Secondary analysis by diagnostic group.

**Methods.** For secondary analyses, cases were classified according to diagnosis at two-year assessment, resulting in “early onset schizophrenia” (EOSz), grouping all schizophrenia spectrum disorders (295.x: schizophrenia and schizoaffective disorder) and “early onset affective disorders” (EOAff) (296.x4: major depressive disorders with psychotic symptoms and bipolar spectrum disorders with psychotic symptoms). The two participants with a diagnosis of psychosis not otherwise specified (298.9) were excluded from this secondary analysis due to its low diagnostic stability (Castro-Fornieles et al., 2011). Statistical analyses were repeated to explore differences in task performance and resting-state fMRI by diagnostic group; and Bonferroni correction was applied in all post-hoc pairwise comparisons.

**Results.** Dividing the sample by diagnostic groups, there were also significant group by condition (X2= 14.7; p = .0006), group (X2= 18.7; p = .0001) and condition (X2= 422.0; p < .0001) effects in the model. Post-hoc analysis revealed significant differences in individuals with EOSz, who showed impaired ToM performance compared to healthy volunteers and to participants with EOAff (ps ≤ .008; Cohen’s d ≥ |1.03|). In contrast, there were no differences between individuals with EOAff and healthy volunteers in the ToM condition, or between groups in the control condition (ps = 1.0). There was no significant effect of gIQ, sex, socio-economic status or age, or for the group by age interaction in the linear regression models. Group differences in intrinsic functional connectivity in cluster 1 (ps ≤ .005) and the group by age interaction in cluster 2 (ps ≤ .001) remained significant for both the EOSz and EOAff groups compared to healthy volunteers [see table A2 and figures A3-A4]. Although the correlation between symptom severity (total score of PANSS) and age of onset did not reach statistical significance in either the subgroup of participants with EOSz (r = -.39; *p* = .13) or with EOAff (r = -.65; *p* = .06), the role of severity of symptoms in the regression models accounting for age was tested. There was no significant effect of severity of symptoms (PANSS: total score and subscales) in ToM performance or functional connectivity (cluster 2) (βs ≤ |.26| ; *ps* > .22) except for the negative subscale of the PANSS on functional connectivity in the medial Prefrontal Cortex (cluster 2) within the contrast including EOSz (β = -.12; *p* = .014), where the effect of age continued to be significant (β = -.40; *p* = .005) [uncorrected *p-* values].

**Table A2.** Socio-demographic and clinical characteristics of the participants and statistical differences between groups.

|  | HV (n=41) | EOSz (n=16) | EOAff (n=9) | *p* value^a^ | Post-hoc  (*Bonferroni*) |
| --- | --- | --- | --- | --- | --- |
| **Socio-demographic** | |  |  |  |  |
| Age (years) | 17.8 (SD=1.6) | 18.0 (SD=1.6) | 17.9 (SD=1.6) | *.654* |  |
| Sex (% female) | 56.1% | 50.0% | 77.8% | *.396* |  |
| Race (% caucasian) | 92.7% | 75.0% | 88.9% | *.172* |  |
| Socio-economic Status | 48.9 (SD=16.0) | 34.7 (SD=13.3) | 44.9 (SD=15.3) | *.008** | HV > EOSz |
| **Clinical variables** | |  |  |  |  |
| Intelligence Quotient | 104.1 (SD=9.8) | 85.8 (SD=12.5) | 101.7 (SD=15.2) | *.0001** | HV = EOAff > EOSz |
| PANSS (total score) | - | 55.9 (SD=13.6) | 41.6 (SD=17.3) | *.008** | EOSz > EOAff |
| - Positive Subscale | - | 10.7 (SD=3.8) | 9.8 (SD=4.1) | *.412* |  |
| - Negative Subscale | - | 19.7 (SD=3.8) | 9.1 (SD=4.6) | *.0006** | EOSz > EOAff |
| - General Subscale | - | 26.1 (SD=8.5) | 22.7 (SD=9.2) | *.113* |  |
| Age of onset | - | 15.9 (SD=1.6) | 15.6 (SD=1.6) | *.497* |  |
| Cumulative Chlorpomazine Equivalents | - | 310827 (141201) | 251035 (216508) | *.126* |  |
| Diagnosis |  | Schizophrenia (n=9)  Schizoaffective disorder (n=7) | Major depressive disorder (n=3)  Bipolar disorders (type I, n=4; no otherwise specified, n=2) |  |  |

Note: HV = Healthy Volunteers; EOSz = Early Onset Schizophrenia; EOAff = Early Onset Affective disorder; PANSS = Positive and Negative Syndrome Scale; ^a^Exact Fisher test or Kruskal-Wallis test for categorical and continuous variables respectively; * *p* < .05

**Figure A3.** Bar graphs representing mean least squares (95% confidence intervals) of performance in the control and experimental conditions of the “Reading-the-Mind-in-the-Eyes” Test (A) and group by age effect on experimental condition (B) for the healthy volunteers (n=41), the early onset schizophrenia spectrum group (n=16) and the early onset of affective spectrum group (n=9).


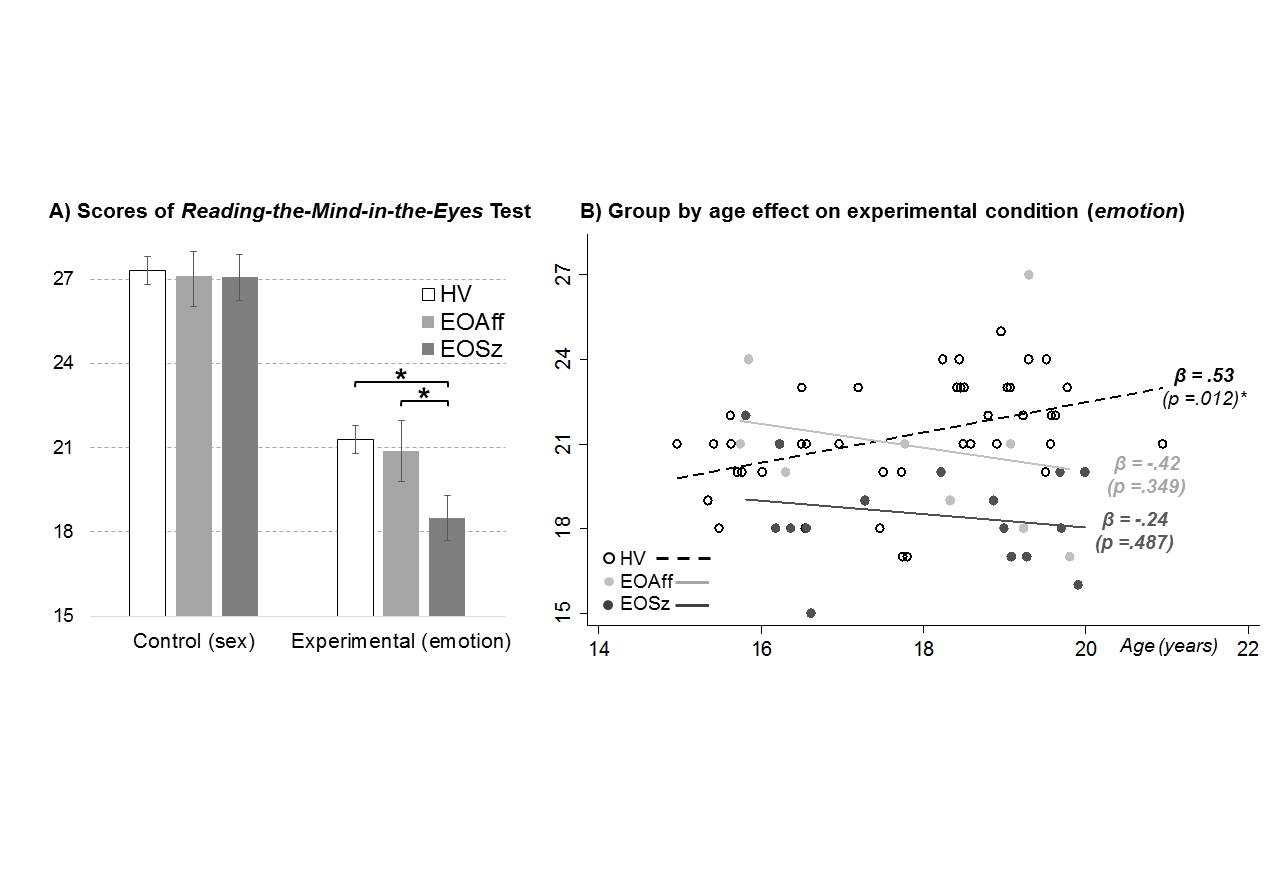


Note: HV = Healthy Volunteers; EOSz = Early Onset Schizophrenia spectrum disorders; EOAff = Early Onset Affective spectrum disorders; * *p* < .05

**Figure A4.** Clusters within the default mode network showing significant group effect (A) and group by age interaction (B) in intrinsic functional connectivity between participants with early onset schizophrenia (n=13), early onset affective disorders (n=9) and healthy volunteers (n=40).


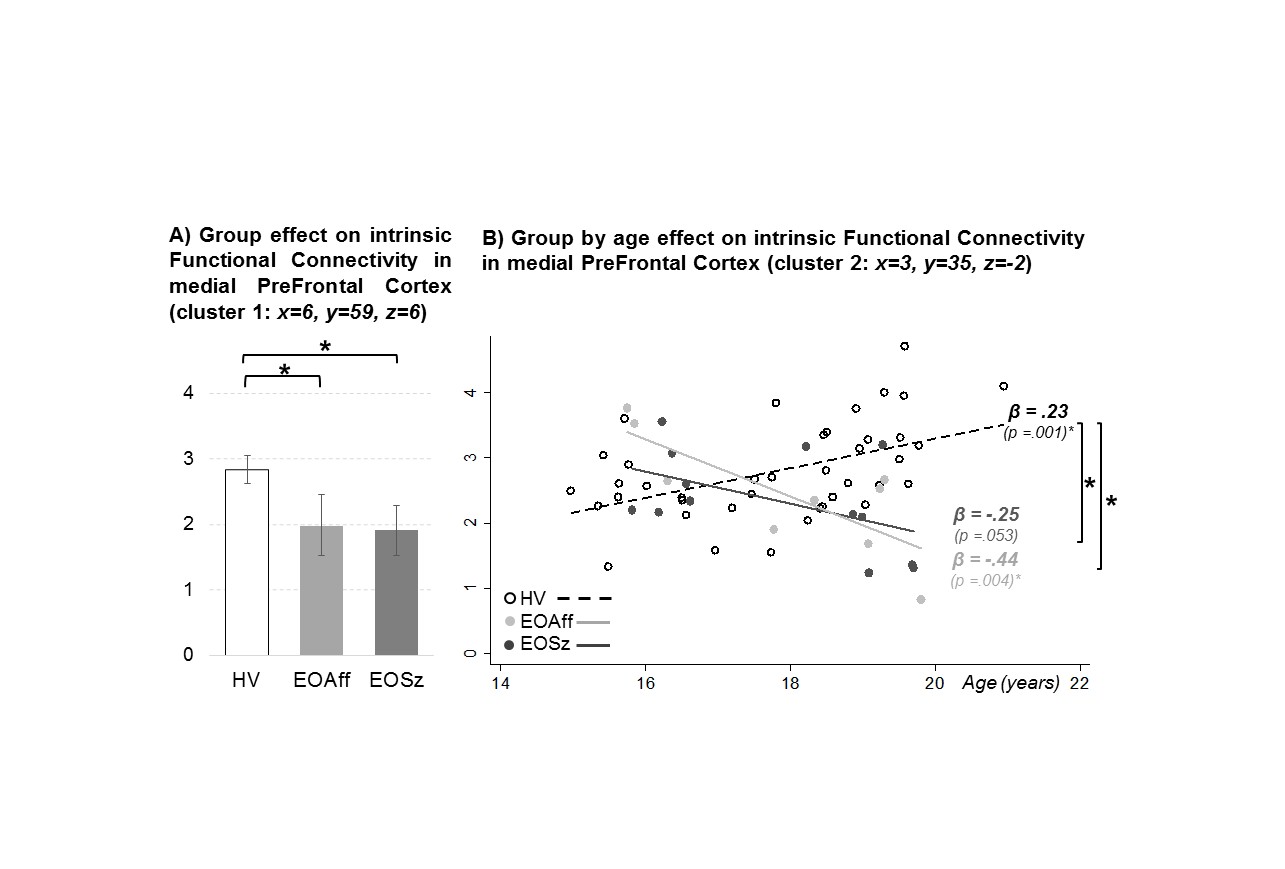


Note: HV = Healthy Volunteers; EOSz = Early Onset Schizophrenia spectrum disorders; EOAff = Early Onset Affective spectrum disorders; * *p* < .05
